# Population pharmacokinetic approach for evaluation of treosulfan and its active monoepoxide disposition in plasma and brain on the basis of a rat model

**DOI:** 10.1007/s43440-020-00115-0

**Published:** 2020-05-30

**Authors:** Dorota Danielak, Michał Romański, Anna Kasprzyk, Artur Teżyk, Franciszek Główka

**Affiliations:** 1 Department of Physical Pharmacy and Pharmacokinetics, Poznan University of Medical Sciences, Święcickiego 6 St, 60-781 Poznań, Poland; 2 Department of Forensic Medicine, Poznan University of Medical Sciences, Święcickiego 6 Street, 60-781 Poznan, Poland

**Keywords:** Blood–brain barrier, Population pharmacokinetics, Alkylating antineoplastic agents, Epoxy compounds

## Abstract

**Purpose:**

Efficacy of treosulfan, used in the treatment of marrow disorders, depends on the activity of its monoepoxy—(EBDM) and diepoxy compounds. The study aimed to describe the pharmacokinetics of treosulfan and EBDM in the rat plasma and brain by means of mixed-effects modelling.

**Methods:**

The study had a one-animal-per-sample design and included ninty-six 10-week-old Wistar rats of both sexes. Treosulfan and EBDM concentrations in the brain and plasma were measured by an HPLC–MS/MS method. The population pharmacokinetic model was established in NONMEM software with a first-order estimation method with interaction.

**Results:**

One-compartment pharmacokinetic model best described changes in the concentrations of treosulfan in plasma, and EBDM concentrations in plasma and in the brain. Treosulfan concentrations in the brain followed a two-compartment model. Both treosulfan and EBDM poorly penetrated the blood–brain barrier (ratio of influx and efflux clearances through the blood–brain barrier was 0.120 and 0.317 for treosulfan and EBDM, respectively). Treosulfan plasma clearance was significantly lower in male rats than in females (0.273 L/h/kg vs 0.419 L/h/kg).

**Conclusions:**

The developed population pharmacokinetic model is the first that allows the prediction of treosulfan and EBDM concentrations in rat plasma and brain. These results provide directions for future studies on treosulfan regarding the contribution of transport proteins or the development of a physiological-based model.

**Electronic supplementary material:**

The online version of this article (10.1007/s43440-020-00115-0) contains supplementary material, which is available to authorized users.

## Introduction

**Fig. 1:**
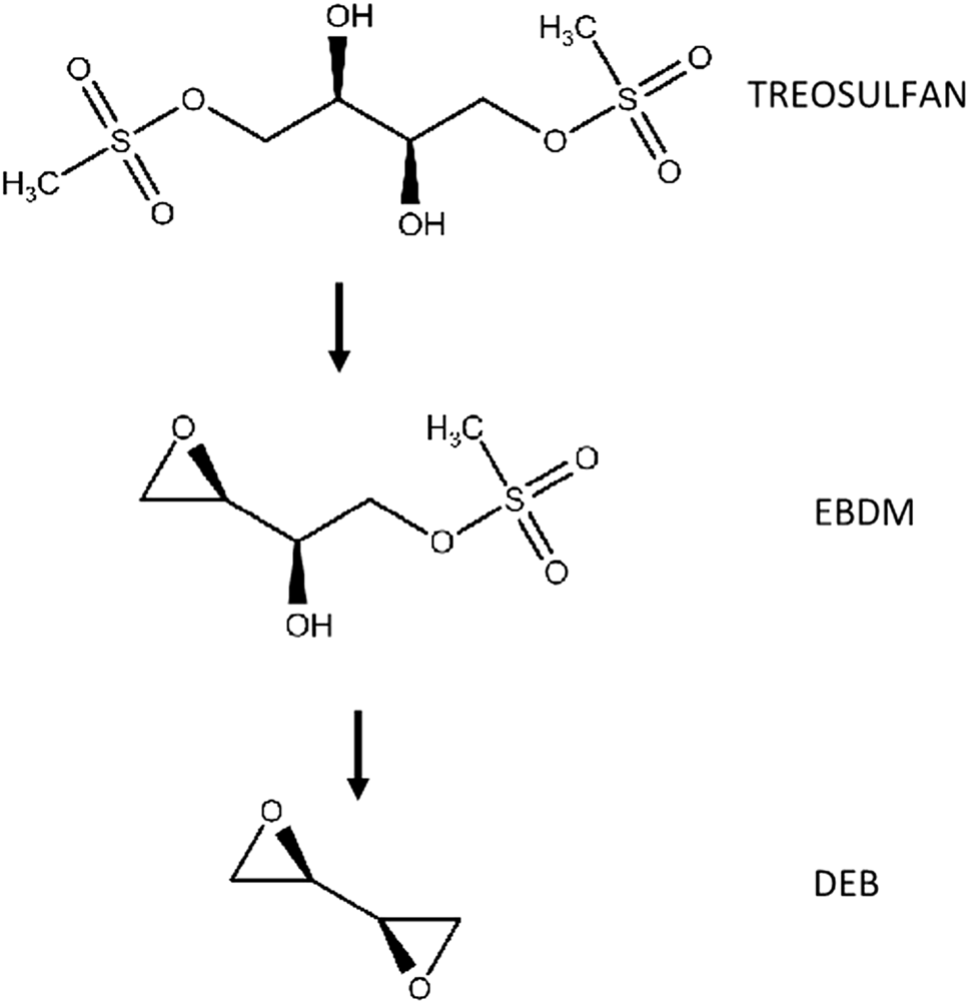
Non-enzymatic transformation of treosulfan to the active epoxide compounds

Treosulfan is a small, polar molecule (molecular weight = 279.29 g/mol) that gains importance in the treatment of marrow disorders. Treosulfan itself does not exert a pharmacodynamic effect, and its cytotoxic properties result from the activity of its transformers. These molecules with one ((2S,3S)-1,2-epoxybutane-3,4-diol-4-methanesulfonate, EBDM) or two epoxide rings ((2S,3S)-1,2:3,4-diepoxybutane, DEB) are formed in a pH and temperature-dependent reaction (Fig. 1). DEB can cross-link DNA strands by alkylating N-7 position in guanine [1, 2].

At first, treosulfan was registered for the treatment of ovarian cancer, under the name Ovastat^®^. In 2004 European Medicines Agency allowed the use of treosulfan in conditioning treatment before hematopoietic progenitor cell transplantation (orphan designation EU/3/04/186). The results of clinical trials indicate that treosulfan is effective and well-tolerated in patients with acute myeloid leukemia [3], chronic granulomatous disease [4] or marrow failure disorders [5]. Also, interim results of a recently completed clinical trial aimed at comparison of treosulfan-based versus busulfan reduced-intensity regimens (NCT 00822393, ClinivalTrials.gov) suggest at least non-inferiority of treosulfan. The research group reported a significantly non-inferior event-free survival in favor of treosulfan (50.4% vs 64.0%, hazard ratio 0.65, *p* = 0.0000164) within 24 months post-transplantation [6]. In this first head-to-head comparison overall survival (71.3% vs. 56.4%; HR 0.61; *p* = 0.0082) and transplantation-related mortality (12.1% vs. 28.2%; HR 0.54; *p* = 0.0201) were also better in treosulfan-treated patients. In June 2019 treosulfan was authorized for marketing by the European Commission as a part of conditioning treatment prior to allogeneic hematopoietic progenitor cell transplantation under the name TRECONDI [7].

The mechanism of transport of treosulfan through the blood–brain barrier is not well known. Several issues hint that there is a need for a further, more thorough investigation of this matter. First, an advantage of treosulfan over busulfan is its low neurotoxicity [4]. Recently conducted disposition studies of a rat model indeed show that the penetration of treosulfan to the brain is limited and the exposure is approximately tenfold lower in the brain in comparison with plasma [8]. Also, the kinetics of treosulfan and EBDM in the brain differed from kinetics in other tissues, with significantly lower elimination constants calculated from a naïve pooled approach in WinNonlin 6.2 software. On average, for treosulfan these values were 0.79 1/h in the plasma, and 0.34 1/h in the brain, while for EBDM—0.79 1/h in the plasma, and 0.59 1/h in the brain, respectively. It is worth mentioning that gender differences in the pharmacokinetics of treosulfan and EBDM were noticed in that animal study. The female rats demonstrated a statistically faster elimination of the two compounds from plasma and organs, including the brain. Moreover, in the females, treosulfan had a lower volume of distribution relative to bioavailability and higher systemic clearance relative to bioavailability than in the males [8].

The low penetration of treosulfan into the central nervous system is advantageous in terms of limited neurotoxicity, but may also pose some challenges. The central nervous system is often seen as a “sanctuary site” because drugs do not usually penetrate into it [9]. The causes of this poor permeation include tight junctions of the endothelial cells, forced transcellular route, and involvement of efflux transporters and metabolizing enzymes [10]. As a result, leukemia may recur as an extramedullary relapse in sites other than the bone marrow [11]. It may present as granulocytic sarcomas and chloromas and the central nervous system is one of the most common sites [11–13].

These results indicate that pharmacokinetics of treosulfan need further investigation, especially in the aspect of penetration into the central nervous system. Therefore the present study aimed to construct a joint population pharmacokinetic model that would describe the pharmacokinetics of both treosulfan and EBDM in plasma and the brain, on the basis of the data gathered from the aforementioned animal-model study.

## Materials and methods

### Study design and sample analysis

**Fig. 2:**
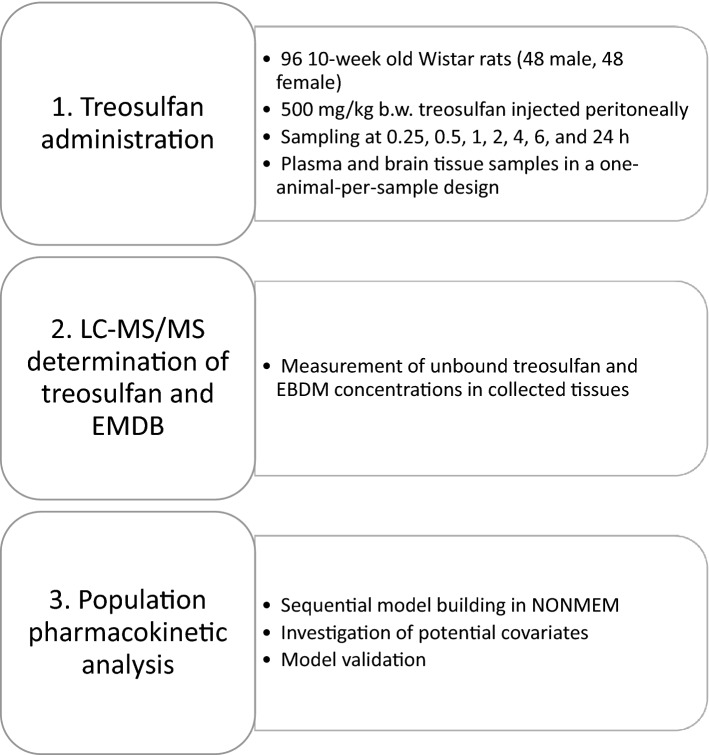
Study outline and workflow

As mentioned before, the present study presents a population pharmacokinetic approach and an in-depth analysis of data gathered in our previously published study, with a focus on plasma and brain concentrations of treosulfan and EBDM [8]. The general outline of the study is presented in Fig. 2.

#### Animals

The study included a total of 96 Wistar rats: 48 male and 48 female. Local Ethics Committee for Experimental on Animals approved the design of the study. All of the procedures were conducted in full compliance with the European Community guidelines, as well as national, ethical, and regulatory principles, per the spirit of Association for Assessment and Accreditation of Laboratory Animal Care (AAALAC). Every possible effort was made to minimize animal suffering.

The animals adapted to the conditions for 2 weeks in standard cages under controlled temperature (22 ± 1 °C), humidity (55 ± 15%), and lightning (12 h light/dark cycle). Rats had ad libitum access to water and a standard commercial feed. On the day of the study, the animals were 10-week old, with a mean bodyweight of 306 ± 25 g and 188 ± 15 g for males and females, respectively.

#### Treosulfan administration

Certified reference material of treosulfan was kindly supplied by medac GmbH (Wedel, Germany). The solution for injection was prepared by dissolving 1000 mg of the drug powder in 20 mL sterile water. A 500 mg/kg b.w. the dose of freshly prepared treosulfan solution was injected intraperitoneally into the lower left quadrant of the abdomen. Before blood and tissue collection, the animals underwent general anesthesia with an intraperitoneal bolus of 50 mg/kg b.w. ketamine (Bioketan, Vetoquinol Biowet, Gorzów Wlkp., Poland) and 10 mg/kg b.w. xylazine (Sedazin, Biowet Puławy, Puławy, Poland).

#### Sample collection

The study followed a one-point per animal design. The samples were collected predose and 0.25, 0.5, 1, 2, 4, 6, and 24 h after the treosulfan injection. From each animal 5 mL of blood was withdrawn *via* heart puncture. The blood was immediately acidified by the addition of 50 µL of 1 M citric acid per 1 mL of blood (P.O.Ch., Gliwice, Poland) to halt ex vivo conversion of treosulfan to EBDM and further transformation of EBDM to DEB. After blood collection, the animals were sacrificed for the collection of organs. Brains were rinsed with 0.9% NaCl, weighed, and mechanically homogenized with 0.05 M citric acid (5 mL/g of the tissue). Full blood and tissue homogenates were centrifuged at 4000 × g for 10 min. Resulting plasma and particle-free supernatants were stored at − 80 °C until further analysis.

#### Sample analysis with an LC–MS/MS method

Treosulfan and monoepoxide were determined with a validated LC–MS/MS method [14]. The samples, spiked with an internal standard (codeine), were deproteinized in ultrafiltration vials with a 30 kDa cutoff point. The ultrafiltrate was injected into an HPLC system either ‘as it was’ or after dilution with 0.25 mM citric acid. The two analytes, treosulfan and EBDM, and codeine separated on a Zorbax Eclipse Plus C18 column, rinsed with a mixture of a formate buffer (pH 4.0) and acetonitrile (95:5, *v*/*v*). The analytes were detected in a multiple reaction monitoring modes, and the electrospray ionization interface operated in a positive mode. Drying gas (nitrogen) flew at a rate of 10 L/min at 300 °C. The mass transitions used for monitoring treosulfan were 296.0 → 183.1 (for quantification), 296.0 → 279.1, and 296.0 → 87.1 (for identification); the mass transition used for monitoring and quantitation of EBDM was 296.0 → 87.1. The corresponding collision energies were 5 eV, 5 eV, 9 eV, and 5 eV, respectively.

Quality control was assured by the use of calibration standards, prepared by spiking a drug-free matrix with known concentrations of the analytical standards. The lower limits of quantitation for treosulfan in plasma and brain homogenate supernatant were 0.22 µM and 0.11 µM, respectively; limits for monoepoxide were 0.93 µM and 0.19 µM, respectively. Concentrations of treosulfan and EBDM in the rat brain were calculated knowing that 1 µM in the brain homogenate supernatant was equivalent to 6 µmol/kg in the brain tissue [8]. Then, concentrations expressed in µmol/kg were recalculated to µM, basing on the average density of the rat brain, equal to 1.04 g/mL of the tissue [15]. Since both treosulfan and EBDM bind weakly to proteins in plasma and brain homogenates (unbound fraction of 0.940–1.092 [16]) we assumed that all the measured concentrations represent free unbound drug.

### Population pharmacokinetic analysis

#### Software

NONMEM software package (version 7.3.0, ICON Development Solutions, Hanover, MD, USA) was used for pharmacokinetic modeling. Diagnostic plots were generated with the R program (version 3.5.1, Foundation for Statistical Computing, Vienna, Austria) and Xpose (version 4.6.1, Uppsala University, Sweden). Perl Speaks NONMEM scripts (PsN, version 4.8.1) [17, 18] were utilized for model diagnosis and validation. The modeling process was managed through a Pirana pharmacometric workbench (version 2.9.8) [19].

#### Methods

Pharmacokinetic parameters in linear models were estimated with a first-order estimation method with interaction (FOCE-I) through an ADVAN5 subroutine. A significant improvement in the model fit was marked by a 3.84 decrease (*p* < 0.05) in the objective function value (OFV) between two nested models. Model fit was also assessed by visual examination of diagnostic plots. For each model following plots were evaluated: individual (IPRED) and population- predicted (PRED) concentrations versus observed concentrations, IPRED and PRED concentrations versus time, weighted (WRES) and conditional-weighted residuals (CWRES) versus predicted concentrations, WRES and CWRES versus time and the distribution of CWRES.

The model was parametrized in terms of clearance (Cl) and distribution volumes (*V*). Unless a defined kinetic constant value was used, the transfer between adjacent compartment was parametrized in terms of intercompartmental clearance (*Q*). The dose of treosulfan was specified in the model as 1797 µmol/kg for each animal. Therefore, all of the distribution volume and clearance parameters are also expressed per kg body weight. The pharmacokinetic parameters were assumed to be log-normally distributed [20], and the interindividual variability (IIV) elements were applied exponentially, as follows:

$${\theta }_{ij}={\theta }_{j}\times {e}^{{\eta }_{ij}},$$

where *θ*_*ij*_ is a value of the *j*-th pharmacokinetic parameter for an *i*-th individual, *θ*_*j*_ is the population parameter estimate, and *η*_*ij*_ is a random variable characterizing IIV, which is normally distributed with mean zero and variance of *ω*^2^.

A potential correlation between IIV elements was evaluated and included if they significantly improved the model.

In one-point per animal designs it is not possible to estimate residual variability, as the information present in the data is insufficient [21]. Therefore, elements associated with this variability were fixed to the value associated with the allowed error of the LC-MS/MS method (15%). Since the precision of determination of very low treosulfan concentrations in brain homogenates was exceeding 15%, the residual variability in this matrix was set to 20%. Also, the quantitation limit of the method was small in comparison to the observed concentrations, and the calibration curves followed a simple *y* = *ax* linear model. Therefore, we decided to employ a proportional error model, as defined by the following equation:

$${C}_{\mathrm{o}\mathrm{b}\mathrm{s}}= {C}_{\mathrm{p}\mathrm{r}\mathrm{e}\mathrm{d}}+ {C}_{\mathrm{p}\mathrm{r}\mathrm{e}\mathrm{d}}\times \varepsilon,$$

where *C*_obs_ and *C*_pred_ are observed and predicted concentrations, and *ε* is a variable associated with proportional residual variability.

*η*-shrinkage was calculated by the following equation:

$$\mathrm{S}\mathrm{h}\mathrm{r}\mathrm{i}\mathrm{n}\mathrm{k}\mathrm{a}\mathrm{g}\mathrm{e}=1- \frac{\mathrm{S}\mathrm{D}(\eta )}{\omega },$$

where SD is a standard deviation of *η* and *ω* is the population model estimate of the standard deviation in *η*.

#### Model development

Following assumptions were made before model development:

1. Pharmacokinetics of treosulfan and EBDM is linear because previously published research showed linear changes of the area under the time-concentration curve (AUC) and maximum concentration (*C*_max_) in plasma up to the total dose of 47 g/m^2^ (1161 mg/kg b.w.) [22];
2. Unbound fraction of treosulfan and EBDM in the plasma and brain homogenate is above 0.94 [16]. Therefore, it is assumed that the measured concentrations are equivalent to the unbound concentrations;
3. Transformation of treosulfan to EBDM is irreversible [23];
4. Transformation rate constant of treosulfan to EBDM in plasma (pH = 7.40) equals to 0.451 1/h [23];
5. The transformation rate constant of treosulfan to EBDM in brain homogenate was calculated according to the following equation [24]:
  $$\mathrm{l}\mathrm{o}\mathrm{g}{k}_{f}=-7.479+0.960\times \mathrm{p}\mathrm{H},$$
  where *k*_*f*_ is the transformation rate constant. It was not possible to measure the exact pH of each brain sample. Therefore it was assumed that it has a pH similar to the intracellular pH in the brain, which is equal to 7.2 [25];
6. Treosulfan can be eliminated either in the unchanged form via the urinary pathway or transformed into EBDM; other pathways of elimination are negligible [26–28];
7. Blood–brain barrier transport was estimated similarly as presented by Borström et al. [29]. It was parameterized in terms of the influx clearance (CL_in_) and the extent of the transport (BBB), according to the following equations:
  $${\mathrm{C}\mathrm{L}}_{\mathrm{i}\mathrm{n}}={k}_{\mathrm{i}\mathrm{n}}\times {V}_{\mathrm{p}\mathrm{l},c},$$
  $${\mathrm{C}\mathrm{L}}_{\mathrm{o}\mathrm{u}\mathrm{t}}={k}_{\mathrm{o}\mathrm{u}\mathrm{t}}\times {V}_{b,c},$$
  $$\mathrm{B}\mathrm{B}\mathrm{B}= \frac{{\mathrm{C}\mathrm{L}}_{\mathrm{i}\mathrm{n}}}{{\mathrm{C}\mathrm{L}}_{\mathrm{o}\mathrm{u}\mathrm{t}}},$$
  where CL_in_ and CL_out_ are influx and efflux clearances, respectively, and *k*_in_ and *k*_out_ represent the transport rate constants between the central plasma and the central brain compartments with the respective volumes of *V*_pl,*c*_, and *V*_*b*,*c*_.

The model was developed sequentially. No preliminary data on the population pharmacokinetic parameters of treosulfan in rats were available. Therefore it was not possible to input previously reported values as fixed. First, only the data on treosulfan plasma concentrations were included, then EBDM plasma concentrations were added, followed by treosulfan brain concentration and finally EBDM brain concentrations. At each step, the models were examined as described in the previous section. The volume parameters of the model that best fitted the observed data were fixed to the estimated values and included in the next step of the analysis. With this approach, along with fixed transformation rate constants, it was possible to avoid problems with the identifiability of clearance parameters.

#### Influence of sex

The influence of sex on pharmacokinetic parameters was investigated. A PsN script scm (stepwise covariate model building from NONMEM model) was utilized. The significance of covariates was evaluated in a “forward-inclusion backward elimination” process. First, the sex was added as a covariate for all of the parameters, and the OFV of the nested models was calculated. The most significant parameter-covariate relationship that was associated with the largest decrease in OFV was retained. The process was repeated until no further relationships were significant (ΔOFV > 3.84, *p* < 0.05). In the second step, the covariates were sequentially removed. A relationship was kept in the model if the OFV increased by more than 6.67 (*p* < 0.01). Only the most significant parameter-covariate relationships were included in the final pharmacokinetic model.

#### Model validation

An internal validation approach was applied because of the limited number of samples for each time point assumed in the study. First, a visual predictive check (VPC) was performed for 1000 simulated observations. 95% prediction intervals (PI) were constructed upon the simulated time-concentration data and compared with the observed data. Second, the bootstrap analysis was performed. 1000 bootstrapped datasets were used for the determination of a median value and 5th–95th confidence intervals (CI) for each parameter included in the model. To better resemble the structure of the original dataset, the resampling procedure was stratified on time points and animal sex. Therefore, each of the bootstrapped sets included at each time point the same number of animals of the same sex, as in the original dataset. Bootstrapped values of pharmacokinetic parameters were compared with the final model estimations.

## Results

### Evaluation of measured treosulfan and EBDM concentrations

Inspection of the measured concentrations revealed that one of the female rats had exceptionally high concentrations of treosulfan in plasma 0.25 h after administration (3059.7 µM). This concentration was almost threefold higher than the average concentration in the rest of the samples (1147.0 µM), and the associated absolute value of CWRES exceeded 5. Therefore we decided to remove the data from this individual from the analysis. Also, samples collected 24 h after the administration had concentrations of the analytes below their respective quantitation limits, and this time point was excluded from the further analysis.

### Development of the final population pharmacokinetic model

**Fig. 3:**
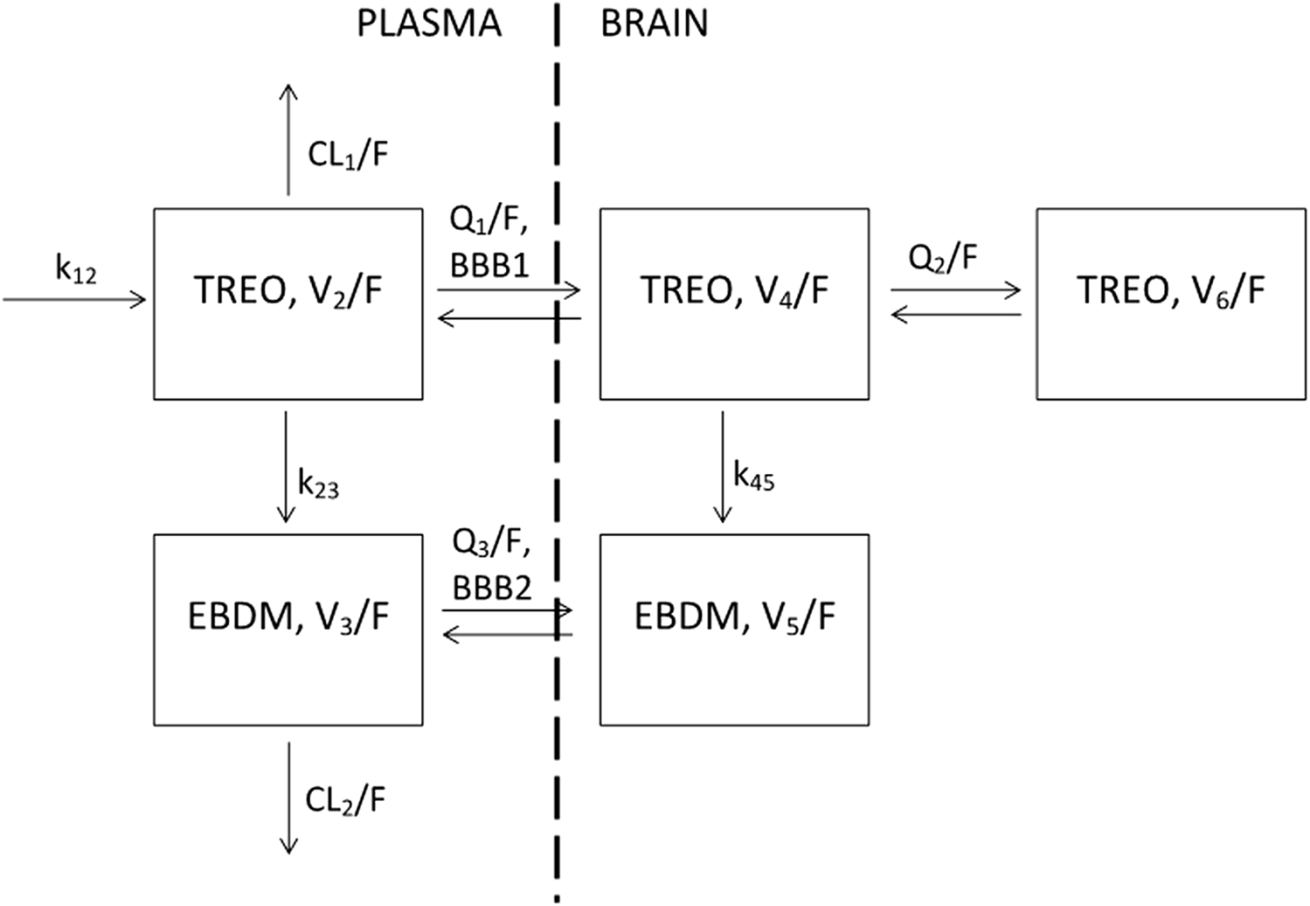
Final structural population pharmacokinetic model

**Fig. 4:**
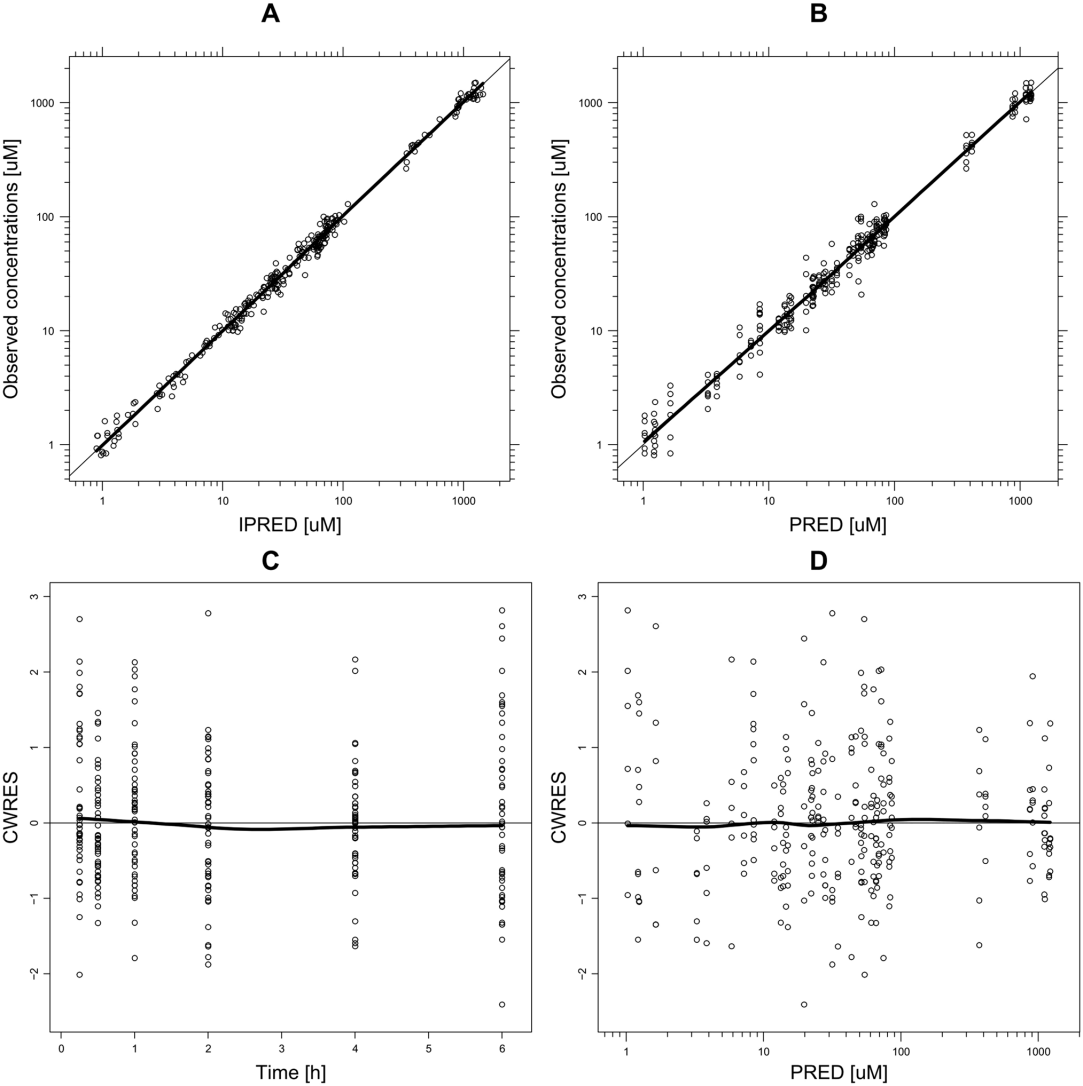
Goodness-of-fit plots for pooled data. Panel **a** illustrates observed concentrations versus individual predicted concentrations (IPRED), panel **b** illustrates observed concentrations versus population predicted concentrations (PRED), panel **c** illustrates conditional-weighted residuals (CWRES) versus time, and panel **d** illustrates CWRES versus PRED. Panels **a** and **b** include a unity line (*y* = *x*; thin black line). On each graph a spline line is included (bold black line)

**Fig. 5:**
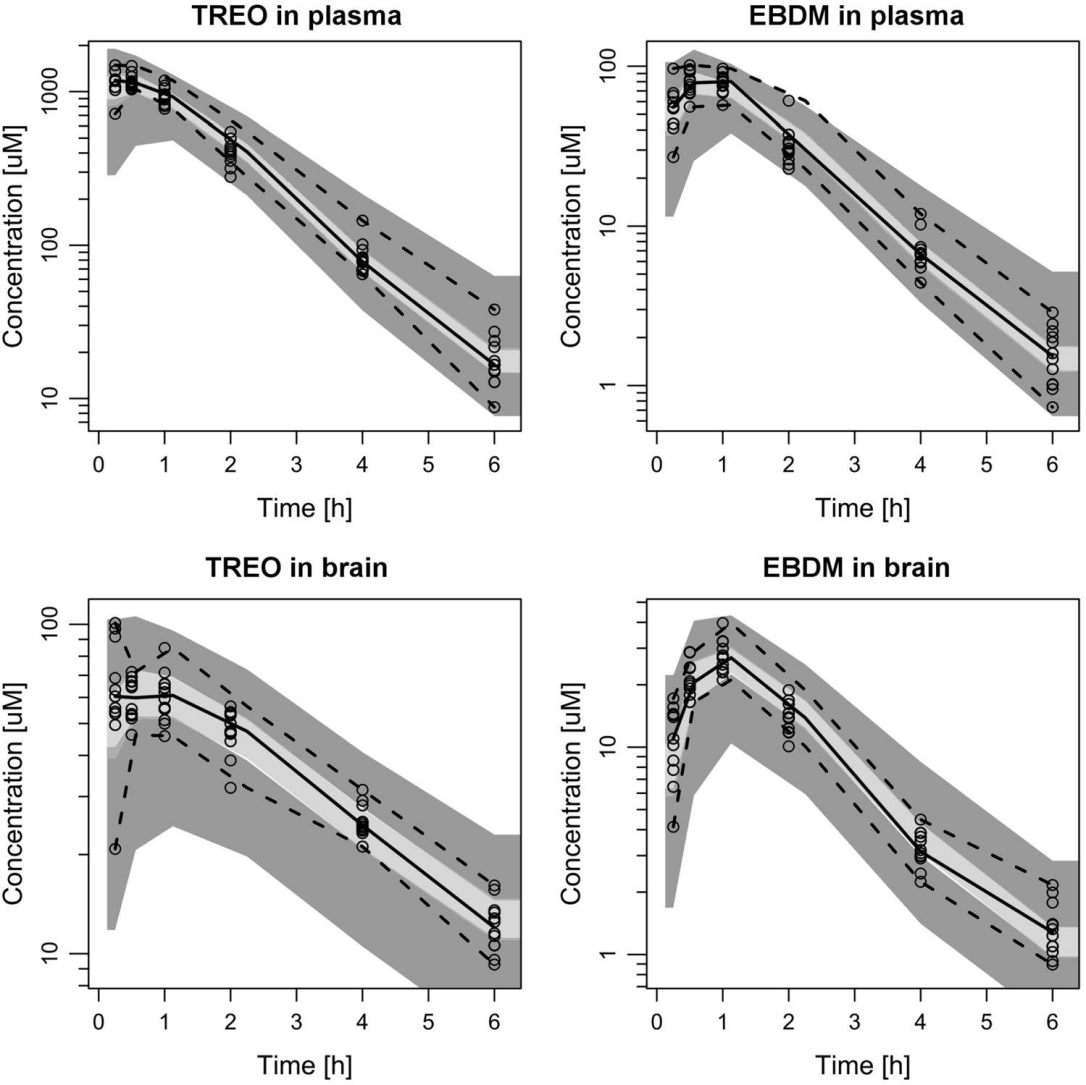
Visual predictive check (VPC) plots for the analyzed concentrations in each tissue. Circles are measured concentrations, solid lines are median, and dashed lines are 5th and 95th percentiles of measured concentrations, light grey areas are 50% interval of simulated data, dark grey areas are 90% interval of simulated data

The final structural model is presented in Fig. 3, while Fig. 4 shows diagnostic plots for the pooled observed data. The IPRED and PRED highly correlate with the observed data. All of the residuals (CWRES) are within ± 3, and no significant trends are visible in the graphs representing CWRES vs time (Fig. 4c) and CWRES vs concentration (Fig. 4d). This implies that all relevant compartments were included in the model and the assumed proportional error model is adequate. The VPC graphs (Fig. 5) show that most of the observed data (93.3%) fall inside the 90% PI. The Supplementary Data file contains detailed goodness-of-fit graphs for each type of observation. The results of the present experiment show that overall, the model predicts well the concentrations of treosulfan and EBDM both in plasma and in the brain. Table 1 presents estimates of the fixed and random effects derived from the final population pharmacokinetic model.

**Table 1:** Estimates from the final pharmacokinetic model and the mean values obtained from the bootstrap analysis

Parameter	Final model estimate (%RSE)	Mean boostrapped estimate (95% CI)
Fixed effects		
*k*_12_ [1/h]	5.12 (17)	5.40 (4.16–6.08)
*V*_2_/*F* [L/kg]	1.03 (fixed)	1.03 (fixed)
CL_1_/*F* [L/h/kg]	0.419 (5)	0.412 (0.386–0.452)
*V*_3_/*F* L/kg]	0.914 (fixed)	0.914 (fixed)
*k*_23_ [1/h]	0.451 (fixed)	0.451 (fixed)
CL_2_/*F* [L/h/kg]	6.24 (3)	6.26 (6.00–6.49)
*Q*_1_/*F* [L/h/kg]	0.0233 (14)	0.0230 (0.021–0.026)
*V*_4_/*F* [× 10^–3^ L/kg]	6.56 (fixed)	6.56 (fixed)
*k*_45_ [1/h]	0.271 (fixed)	0.271 (fixed)
*V*_5_/*F* [× 10^–3^ L/kg]	6.56 (fixed)	6.56 (fixed)
*Q*_2_/*F* [L/h/kg]	0.304 (19)	0.301 (0.251–0.356)
*V*_6_/*F* [L/kg]	0.364 (fixed)	0.364 (fixed)
*Q*_3_/*F* [L/h/kg]	0.0122 (10)	0.0122 (0.011–0.014)
BBB_1_	0.120 (5)	0.120 (0.116–0.124)
BBB_2_	0.317 (5)	0.317 (0.303–0.332)
COV_CL-MALE_^a^	− 0.146	− 0.144 (− 0.223– − 0.068)
IIV [%]^b^		
*ω*_k12_	69 (17)	62.3 (45.0–90.1)
*ω*_CL_	10 (35)	12.5 (9.4–17.0)
Residual error		
For TREO and EBDM in plasma	0.15 (fixed)	0.15 (fixed)
For TREO and EBDM in brain tissue	0.20 (fixed)	0.20 (fixed)

*RSE* relative standard error, *CI* confidence interval

^a^CL_1-MALE_/*F* = CL_1_/*F* + CL_1_/*F* * COV_CL-MALE_

^b^%CV = (SQRT(EXP(OMEGA(*N*)) − 1))*100%

#### Absorption of treosulfan

Peritoneal administration of treosulfan allowed rapid absorption of the drug (*k*_12_ = 5.12 1/h), but the IIV of this parameter is large and exceeds 50% (Table 1). Several models for this type of administration were tested: an iv bolus model, a first-order absorption, and a zero-order infusion. The data indicated that it resembled most closely a first-order extravascular absorption, rather than an intravascular infusion (a zero-order absorption) or an intravascular bolus. Therefore, the clearance and volume parameters were adjusted by bioavailability (*F*).

#### Prediction of treosulfan and EBDM concentrations in plasma

One compartment model sufficiently described changes in the concentrations of treosulfan and EBDM in plasma. Addition of a second tissue compartment improved the model fit neither for treosulfan nor EBDM (increase in the OFV by 0.9 and 160.1 for treosulfan and EBDM, respectively).

#### Prediction of treosulfan concentrations in brain

As presented in the VPC graphs (Fig. 5), changes of treosulfan concentrations in the brain are most varied, especially within the first 0.5 h after administration. Also, the concentrations achieve peak values quickly and without any distinctive maximum, as opposed to the concentrations of EBDM in the brain, which reach a maximum at 1 h after administration. Therefore, a second compartment (*V*_6_/*F*) had to be included to describe the pharmacokinetics of treosulfan in brain adequately. It significantly improved the fit of the model and lowered the OFV (ΔOFV = − 126.04 between the two discussed models). The estimated value of *V*_4_/*F*, which is associated with the concentrations of treosulfan in the brain homogenate, was small compared with the other compartments and equalled approximately 3 mL/kg. This value was similar to the average volume (6.56 mL/kg) of extravascular space in rat brain [30]. Therefore, we decided to fix this parameter to the physiological value, to better evaluate the clearance parameters. Although the imputation of fixed values is only an approximation, it has some advantages in complicated models like the one presented in this paper. We noticed that the model with fixed volume values was more stable than the models in which the distribution volumes were estimated, and both minimization and covariance steps were successful.

#### Prediction of EBDM concentrations in the brain

The analogous approach as described in the previous section was applied to EBDM. The distribution volume of EBDM in the brain (*V*_5_/*F*) was also set to the physiological value of 6.56 mL/kg because in the first estimation *V*_5_/*F* was very similar (5.6 mL/kg). We tested models that assumed exclusive formation of EBDM from treosulfan in the brain tissue, but the models that well fitted the data included both the transport of EBDM across the blood–brain barrier and its formation from treosulfan. VPC graphs (Fig. 4) for EBDM in the brain show that the observed concentrations might follow two-compartment pharmacokinetics, similar to treosulfan. The addition of a peripheral brain compartment for EBDM decreased the OFV by 26.126. However, the confidence intervals and standard errors calculated in the bootstrap analysis were large, and the more complex model unreliably predicted the parameters. For the peripheral brain compartment, the standard error of the distribution volume was 228.1%, while for the associated intercompartmental clearance it was 120.3%. Therefore we retained a simplified model, with only a central brain compartment for EBDM.

#### Interindividual variability of estimated pharmacokinetic parameters

During the model refinement step we inspected the IIV values, their standard errors, and shrinkage. We excluded from the model the IIV parameters that could not be adequately and precisely estimated from the model (e.g. RSE > 100%) or had a high shrinkage. After removing such a parameter, we inspected the potential changes in the OFV, the diagnostic plots, and if the values for other estimated parameters were reasonable. The final model includes IIV estimates for only *k*_12_ and CL_1_/*F*. The calculated shrinkage is 27% and 56%, respectively. Shrinkage above 30% may influence the power of diagnostics for individual predicted parameters and concentrations [31]. However, removing the IIV on CL_1_/*F* negatively impacted the model fit and stability. High shrinkage might be a result of the type of data available for model development that is a one-point-per-animal experiment. The condition number (calculated from the ratio of the largest and the smallest eigenvalues) of the final model was 44.5, and it indicates that the model is not over-parametrized or ill-conditioned.

#### Influence of sex on the pharmacokinetics of treosulfan and EBDM

**Fig. 6:**
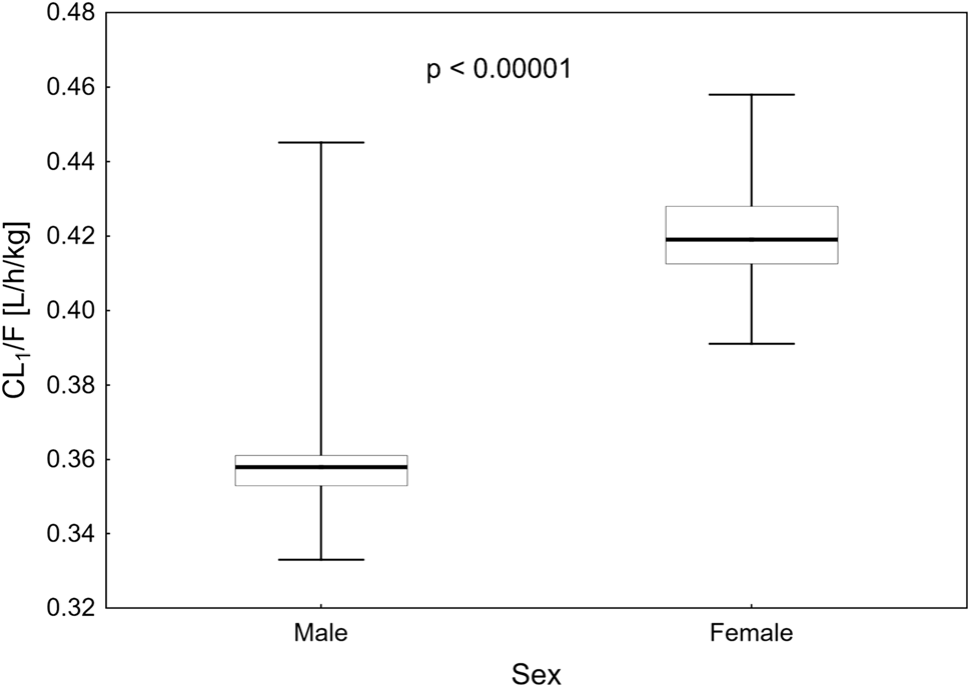
Dependence of systematic treosulfan clearance (CL_1_/*F*) on sex. Box areas represent 25th–75th percentiles with a thick line as a median, whiskers as the minimum and maximum values, and the associated *p* value

Sex was the only covariate for which we could evaluate an influence on treosulfan and EBDM pharmacokinetics. In the forward-inclusion step of the stepwise covariate modeling method, the inclusion of sex significantly lowered the values IIV of plasma clearances of treosulfan and EBDM (Fig. 6). However, only the relation between treosulfan plasma clearance (CL_1_) and sex remained significant after the backward-elimination process. The model that included the slower elimination of treosulfan from plasma in males had the OFV lower by 9.50 (1403.2 without the covariate vs 1393.7 with the covariate, *p* = 0.002), and the interindividual variability of CL_1_ decreased by 7.9%.

### Blood–brain transport of treosulfan and EBDM

Blood–brain barrier parameters for treosulfan and EBDM (BBB_1_ and BBB_2_) were below 1 (Table 1). These results indicate that the efflux of both compounds is greater than their influx into the brain tissue. Evaluation of the transport through the blood–brain barrier revealed that this process is most probably bilateral because the models with one-way blood–brain transport were ill-fitted. We also tested, whether an additional elimination of treosulfan or EBDM from the brain compartments was possible. However, available data did not support the models that assumed such a process.

## Discussion

In this study, a joint parent-metabolite population pharmacokinetic model was developed for the first time that allows the prediction of treosulfan and EBDM concentrations in plasma and brain homogenates in rats. We confirmed that treosulfan poorly penetrates the blood–brain barrier and the efflux of the drug is almost tenfold greater than the influx. EBDM is also efficiently removed from the brain tissue, but to a less extent than treosulfan. We also found that the elimination of treosulfan from the plasma is slower in male rats.

In the present study, we utilized nonlinear-mixed effect methods, which are a gold standard for building population pharmacokinetic models. It has several advantages over a naïve pooled approach that was used in a previous paper [8]. First, it allows a more detailed exploration of the model structure and possible mechanisms underlying variability in drug pharmacokinetics. Also, it is possible to estimate between-subject variability. In our model, changes of treosulfan concentrations in plasma followed first-order kinetics and the addition of a second, tissue compartment did not improve the model. Most of the available literature on treosulfan states that a two-compartment model best describes the pharmacokinetics of this drug in patients [27, 28, 32, 33]. This difference might stem from a small number of time points in the present animal study. Blood and tissues were sampled up to 6 h after treosulfan administration, but four points only were post-*C*_max_. This number might be insufficient to support a more complex two-compartment model, as it is difficult to discriminate between fast and slow disposition phases.

The distribution volume of treosulfan (*V*_2_/*F*) estimated with the final model was similar to the values reported by Romański et al. (0.96 ± 0.06 L/kg in female rats and 1.02 ± 0.07 L/kg in male rats) [8]. The authors reported that the elimination constant of treosulfan depended on sex and that this difference might be caused by lower distribution volume and higher clearance in female rats. In the present study, sex was a significant covariate for systemic clearance only. Interestingly, none of the studies on a human population found sex as a significant covariate on treosulfan pharmacokinetics [32–35].

Gender differences in the expression of organic anion transporters (OATP) might explain the differences observed in our study. Expression of the gene encoding OATP1 transporter, localized at the apical plasma membrane of the kidney, is much higher in female rats; as a result, systemic clearance of OATP1 substrates, such as zenarestat, is elevated [36]. Another transporter, OATP2, is also more strongly expressed in female rats [37]. These gender differences are caused by sex hormones, as estrogen and progesterone upregulate OATP2 expression, and androgens inhibit it [37]. OATP expression might also significantly decrease with age [38]. To our knowledge, no study reported the involvement of OATP in the pharmacokinetics of treosulfan. Therefore, further studies have to be conducted to confirm or reject this hypothesis and to investigate the potential impact on humans.

Several studies provide data on the blood–brain transport of treosulfan and EBDM. In an in vitro study, Linz et al. found that transport of treosulfan in the apical-to-basolateral direction (influx) is lower than the basolateral-to-apical (efflux), with permeability values of 1.6% and 3.0%, respectively [39]. The authors have also calculated a small logBB value (− 1.98), upon the ratio of treosulfan concentrations in brain and plasma. Another study showed that the permeability of treosulfan into the brain tissue depends on age [16]. The study involved juvenile and young adult rats. The brain/plasma ratio of AUC of unbound treosulfan was higher in younger animals—0.14 in juvenile male and 0.17 in juvenile female rats, compared with 0.10 in young adult male and 0.07 in young adult female rats. A similar value (0.1 brain/plasma AUC ratio) presented in a paper that gave rise to the present experiment confirms poor penetration of treosulfan into the brain [8]. In the present study, the blood–brain penetration for treosulfan was 0.120. It was parameterized in terms of clearance ratio, rather than an AUC ratio or a concentration ratio, which might be a source of the difference in the values. Poor penetration of treosulfan is almost counter-intuitive. The molecule fulfills all of the Lipiński’s rule of 5 criteria—its molecular weight is lower than 500, it has fewer than five hydrogen-bond donors and fewer than ten hydrogen-bond acceptors, and its octanol–water partition coefficient (log *P*_*o*/*w*_) is less than 5 [40]. Also, the pharmacokinetic profile of treosulfan in the brain, with relatively high concentrations obtained shortly after administration, suggests that this drug rapidly crosses the blood–brain barrier. At the same time, efflux pumps, such as *P*-glycoprotein, multidrug resistance-associated protein or even OATP, might effectively remove treosulfan from the brain. However, no study so far evaluated the involvement of these transporters in treosulfan pharmacokinetics. Also, the saturability of the process was not assessed. Another finding from the study is a two-compartment disposition of treosulfan in the brain. The distribution volume of the peripheral brain compartment was over 50-fold higher than the volume of the central one. Results from a previous study indicate that for small compounds two compartments may exist in the brain—one that equilibrates rapidly, and the second one that equilibrates for up to 30 min [41]. The authors of this study used tracers that are not taken up into erythrocytes, contrary to treosulfan. Therefore, this implication has to be approached with caution.

The permeability of EBDM through the blood–brain barrier was almost threefold larger than treosulfan (0.317 vs 0.120). It could be explained by lower hydrophilicity of EBDM (log *P*_*o*/*w*_ = − 1.18 for EBDM vs. log *P*_*o*/*w*_ = − 1.58 for treosulfan, respectively) [40]. Also, an additional elimination of EBDM from the brain compartment was insignificant, as its addition to the model did not improve it. It might be surprising, because EBDM transforms to DEB (in vitro *k*_EBDM → DEB, pH = 7.4, *t* = 37 °C_ = 0.206 1/h) and undergoes hydrolysis (in vitro *k*_hydrolysis, pH = 7.4, *t* = 37 °C_ = 0.0269 1/h) [23]. It might indicate that epoxy-transformation and hydrolysis might not have a strong impact on the overall clearance of EBDM. In vivo, specific enzymes—glutathione *S*-transferase and epoxide hydrolase—are involved in the elimination of epoxides. One may hypothesize that the activity of these enzymes, particularly in the liver, contributes to a high systemic clearance of EBDM. Glutathione *S*-transferase also is present in the brain and may play a role in the observed low exposure of EBDM in this organ. Higher expression of this enzyme in endothelial cells and astrocytes may contribute to the resistance to some antiepileptic drugs [42]. Soluble epoxide hydrolase is also present in both rat and human brain and participates in the regulation of cerebral blood flow, and arachidonic and linoleic acid epoxides utilization [43, 44]. However, up to now, no study on the potential impact of the activity of these enzymes on the pharmacokinetics of treosulfan was published.

The limitations of this study also have to be noted. The sampling protocol was destructive, and each data point represents a single individual. Therefore random effects had to be fixed to the error associated with the analytical method. Estimation of random effects could be possible if treosulfan and EBDM concentrations were measured via a microdialysis method and a full pharmacokinetic profile would be provided for each animal. Another advantage of the microdialysis approach would be to apply a prolonged and denser sampling protocol without sacrificing an animal for each data point. Also, the exact pH of brain homogenates was unknown, and the value of transformation constant had to be approximated. Another aspect is the age uniformity. As earlier described, the penetration of treosulfan and EBDM is higher in younger animals. Hence the age might be a significant covariate of BBB parameters.

The results of the present study provide several implications and directions for future studies on treosulfan. If OATPs participate in treosulfan transport via cell membranes, genetic polymorphisms of these proteins might significantly influence the pharmacokinetics of treosulfan or partially explain its variability. Some known polymorphisms in genes encoding OATPs have an established, clinically relevant impact on the safety of drugs. For example, the presence of 512C>T variant in *SLCO1B1* gene (rs4149056) corresponds with decreased clearance of methotrexate and occurrence of statin-induced myopathy [45, 46]. Also, activities and rates of enzymes possibly involved in EBDM utilization in the brain are worth investigating, as they may play a protective role in terms of neurotoxicity. Besides, investigation of permeation of treosulfan and EBDM may help to understand the mechanism of action and role of treosulfan in stabilization or improvement of patients with active secondary progressive multiple sclerosis [47]. Finally, a physiologically-based pharmacokinetic model could be established, to bridge the gap between data collected from animals and humans, both children and adults. However, the development of such a model will provide a challenge, as for an accurate prediction, not only blood flow in organs and enzyme activities have to be known, but also pH and temperature conditions, as the conversion of treosulfan to its epoxides is non-enzymatic.

## Conclusions

The developed population pharmacokinetic model allows accurate prediction of treosulfan and EBDM concentrations in rat plasma and brain homogenates. The systemic clearance of treosulfan is significantly lower in male rats. The efflux of treosulfan and EBDM through the blood–brain barrier markedly exceeds their influx into the brain.

## Electronic supplementary material

Below is the link to the electronic supplementary material.

Supplementary file1 (DOCX 312 kb)
