# Correction to: Population pharmacokinetic approach for evaluation of treosulfan and its active monoepoxide disposition in plasma and brain on the basis of a rat model

**DOI:** 10.1007/s43440-020-00144-9

**Published:** 2020-08-12

**Authors:** Dorota Danielak, Michał Romański, Anna Kasprzyk, Artur Teżyk, Franciszek Główka

**Affiliations:** 1 Department of Physical Pharmacy and Pharmacokinetics, Poznan University of Medical Sciences, Święcickiego 6 St, 60-781 Poznań, Poland; 2 Department of Forensic Medicine, Poznan University of Medical Sciences, Święcickiego 6 Street, 60-781 Poznań, Poland

## Correction to: Pharmacological Reports 10.1007/s43440-020-00115-0

The original version of this article, published on 30 May 2020 contained a mistake.

The authors regret that some of the constants mentioned throughout the article were expressed in an incorrect unit, which is “L/h” instead of “1/h”. These include all of the first-order constants: k_12_, k_23_, k_45_, elimination constants of treosulfan and EBDM from plasma and the brain, transformation rate constant of treosulfan to EBDM, transformation rate constant of EBDM to DEB, and hydrolysis constant of EBDM.

The original article has been corrected.

