# Supplementary material for: Population pharmacokinetic approach for evaluation of treosulfan and its active monoepoxide disposition in plasma and brain on the basis of a rat model
Source: Pharmacol Rep. 2020 May 30;72(5):1297–309. doi: 10.1007/s43440-020-00115-0 (PMC7550288; doi:10.1007/s43440-020-00115-0)

Supplementary Data File 1

Goodness-of-fit diagnostic plots for treosulfan and EBDM in plasma and brain. CMT:2 stands for concentrations of treosulfan in plasma, CMT:3 stands for concentrations of EBDM in plasma, CMT:4 stands for concentration of treosulfan in brain homogenates, and CMT:5 stands for concentrations of EBDM in brain homogenates.

Graphs show as follows: observed concentrations versus individual predicted concentrations (IPRED), observed concentrations versus population predicted concentrations (PRED), conditional-weighted residuals (CWRES) versus time, and CWRES versus PRED. On each graph a green spline line is included.


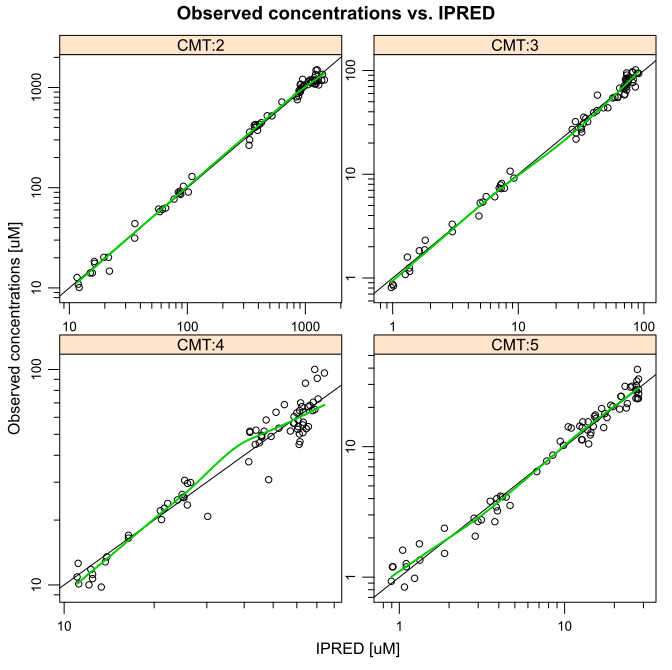


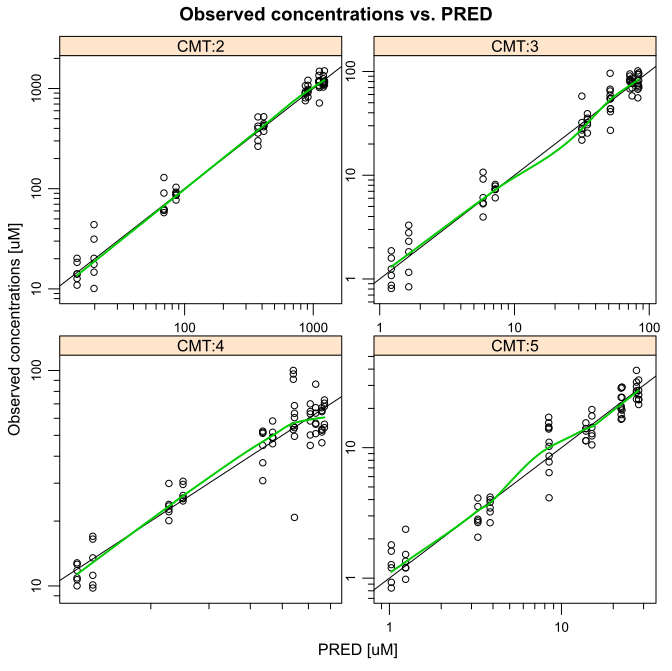


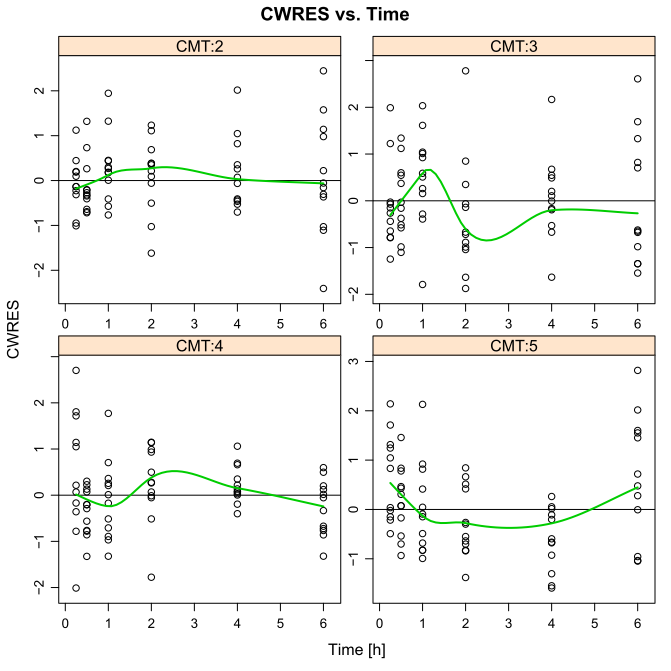

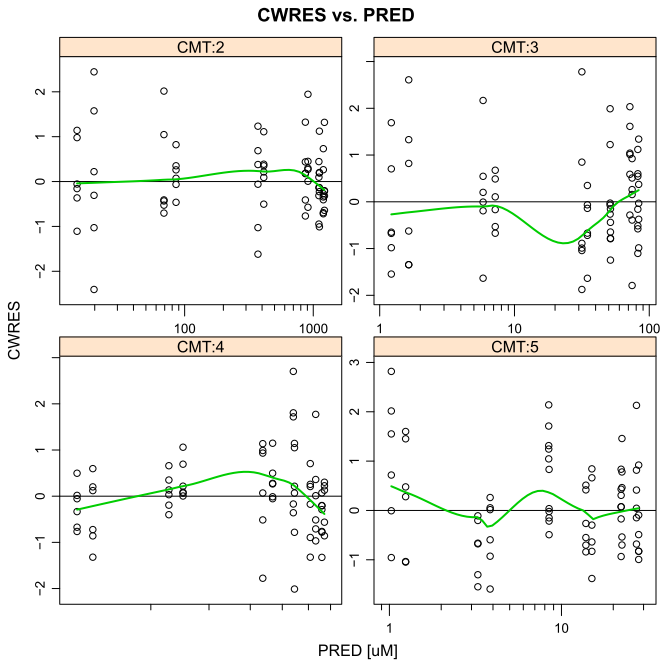

Supplement: Supplementary file 1 — Supplementary file1 (DOCX 312 kb) [file 43440_2020_115_MOESM1_ESM.docx]
